# Retractions in cancer research: a systematic survey

**DOI:** 10.1186/s41073-017-0031-1

**Published:** 2017-05-12

**Authors:** Anthony Bozzo, Kamal Bali, Nathan Evaniew, Michelle Ghert

**Affiliations:** 1 Division of Orthopaedic Surgery, Department of Surgery, McMaster University, 1200 Main Street West, 4E15, Hamilton, ON L8N 3Z5 Canada; 2 Hamilton Health Sciences, Juravinski Hospital and Cancer Center, 711 Concession Street, Level B3 Surgical Offices, Hamilton, ON L8V 1C3 Canada; 3 Hamilton General Hospital—5N Orthopedic Offices, 237 Barton St E, Hamilton, ON L8L 2X2 Canada

**Keywords:** Cancer, Cancer research, Oncology, Oncology research, Research ethics, Retraction, Retractions

## Abstract

**Background:**

The annual number of retracted publications in the scientific literature is rapidly increasing. The objective of this study was to determine the frequency and reason for retraction of cancer publications and to determine how journals in the cancer field handle retracted articles.

**Methods:**

We searched three online databases (MEDLINE, Embase, The Cochrane Library) from database inception until 2015 for retracted journal publications related to cancer research. For each article, the reason for retraction was categorized as plagiarism, duplicate publication, fraud, error, authorship issues, or ethical issues. Accessibility of the retracted article was defined as intact, removed, or available but with a watermark over each page. Descriptive data was collected on each retracted article including number of citations, journal name and impact factor, study design, and time between publication and retraction. The publications were screened in duplicated and two reviewers extracted and categorized data.

**Results:**

Following database search and article screening, we identified 571 retracted cancer publications. The majority (76.4%) of cancer retractions were issued in the most recent decade, with 16.6 and 6.7% of the retractions in the prior two decades respectively. Retractions were issued by journals with impact factors ranging from 0 (discontinued) to 55.8. The average impact factor was 5.4 (median 3.54, IQR 1.8–5.5). On average, a retracted article was cited 45 times (median 18, IQR 6–51), with a range of 0–742. Reasons for retraction include plagiarism (14.4%), fraud (28.4%), duplicate publication (18.2%), error (24.2%), authorship issues (3.9%), and ethical issues (2.1%). The reason for retraction was not stated in 9.8% of cases. Twenty-nine percent of retracted articles remain available online in their original form.

**Conclusions:**

Retractions in cancer research are increasing in frequency at a similar rate to all biomedical research retractions. Cancer retractions are largely due to academic misconduct. Consequences to cancer patients, the public at large, and the research community can be substantial and should be addressed with future research. Despite the implications of this important issue, some cancer journals currently fall short of the current guidelines for clearly stating the reason for retraction and identifying the publication as retracted.

## Background

The retraction of a scientific publication indicates that its findings are invalid and should not influence future research or clinical practice. Several types of research misconduct warrant the retraction of a scientific paper. These include plagiarism, duplicate publication, fraud, authorship issues, ethical issues, and error [1–3].

It is now well documented that the proportion of published studies that are being retracted from the scientific literature is rapidly increasing [4]. One study found that while the number of studies published annually grew by 44% from 2001 to 2010, the number of annual retractions grew by 1000% during the same time frame [5]. A study by Grieneisen et al. found that the number of annual retractions, adjusted for number of publications, increased by a factor of 11.06 over this 10-year period [6]. Retractions are a worldwide phenomenon as authors from multiple countries of origin have been found to be involved in research misconduct [7]. The consequences to authors of retracted studies can be quite severe. If reprimanded by the Office of Research Integrity (ORI), authors have been found to subsequently experience a median decrease of 91.8% in academic output and significant declines in research funding [8].

While the successful identification of erroneous data is considered by some to by an important advance in science [9], the significant cost of retracted publications to the cancer research community must be considered. Although retracted research represents less than 1% of all NIH funding, it has accounted for over $58 million of direct NIH funding over a 20-year period [8]. The NIH budget has a large focus on cancer research which received $5.8 billion USD of NIH funding in 2012, the most of any disease category [10].

Two of the top ten most cited retracted papers are in cancer research and the propagation of invalid findings and can have deleterious effects on cancer patient care [11, 12]. The increasing rate of retracted publications in the scientific literature is an important emerging phenomenon of which clinicians and evidence users of cancer research should be aware. The objectives of this study were to identify the frequency and reasons for retraction of cancer publications and to determine how retracted articles are handled by journals in the cancer field.

## Methods

We performed a systematic survey of retracted articles in the entire corpus of cancer research [13]. We conducted this study according to the relevant guidance from the Cochrane Handbook for Systematic Reviews of Interventions [14], and we report according to the relevant guidance from the Preferred Reporting Items for Systematic Reviews and Meta-Analyses statement [15].

### Eligibility criteria

The inclusion criteria were (1) retracted studies on any cancer topic, (2) all study designs (3) human, animal, and basic science studies. We define basic science as any non-clinical cancer research. Exclusion criteria included (1) retracted papers on topics unrelated to cancer research and (2) retracted articles unavailable in English. In instances of duplicate retractions for manuscripts published in two journals, only the first retracted publication was considered for further analysis.

### Identification of retracted publications

We searched MEDLINE (1946 to present), Embase (1974 to present), and The Cochrane Library (no date limit) on August 23, 2015, for retracted journal articles. In MEDLINE and Embase, the MeSH heading “neoplasm” and all subheadings were used. In the MEDLINE database, we made use of their specific filter for retracted articles and thus did not require retraction-related keywords or MeSH terms. Embase does not have a similar filter thus the keywords “retraction” and “retracted article” were included in the search strategy. The operator “retract*” was used in the Cochrane library search. We used Embase and MEDLINE since research has shown these databases to be interchangeable with Scopus and others [16]. A website dedicated to the archiving of retracted scientific papers, www.retractionwatch.com, was hand searched for additional retracted publications. Table 1 contains the full search strategy.

**Table 1:** Search strategy

MEDLINE	Embase	Cochrane
1. cancer.mp or exp Neoplasms	1. cancer.mp or exp Neoplasm	1. “cancer” in title, abstract, OR as keyword
2. Retracted Publication/	2. Retracted article/	2. “oncology” in title, abstract, OR as keyword
3. 1 AND 2	3. Retraction/	3. retract* anywhere in text
	4. 2 OR 3	4. 1 OR 2
	5. 1 AND 4	5. 3 AND 4

Two reviewers independently screened all titles and abstracts and then screened the full texts of potentially eligible studies for final inclusion. All discrepancies were resolved by consensus or consultation with a senior author.

### Data extraction

Two reviewers extracted in duplicate all relevant data from the first 10% of included studies in order to calibrate the data extraction (AB, KB). Thereafter, the two reviewers extracted data from the remaining studies with one reviewer assigned to each study. The extracted data were stored in an electronic database and included author name, country of origin of corresponding author, year of publication, year of retraction, number of citations, journal name and impact factor, study design, reason for retraction, and accessibility of the retracted article. The impact factor of the journal and the number of citations of each retracted article at the time that we performed the database search were obtained by searching Web Of Science (http://apps.webofknowledge.com.libaccess.lib.mcmaster.ca/). We included JIF as a variable because others have found a strong correlation between frequency of retraction and JIF [17]. To assess the possibility of a trend, we determined the total number of cancer publications and retractions appearing in the MEDLINE database in each year from 2000 to 2010.

Whenever possible, we used the journal’s official retraction statement to determine the reason for retraction. The reasons for retraction that consistently appear in the retraction literature include [7]: plagiarism, duplicate publication, fraud, error, authorship issues, and ethical issues. While plagiarism and duplicate publication are self-explanatory, the category of fraud includes issues such as data, image or figure manipulation, and tampering of the peer review process. Error on the part of the authors includes using the use of incorrect cell lines, data errors, and inability to reproduce results. An example of an authorship issue is inclusion of authors unaffiliated with the study. Ethical issues include lack of prior ethics approval for the study or failure to acquire patient informed consent.

In order to tabulate how a journal handled a retraction, the retracted article was classified as “intact” if it could be accessed online without any alteration (such as a watermark) from the original publication. The article was classified as “removed”; if the original webpage for the article and a retraction notice were found, but the article itself was removed and no PDF was available for download. The article was classified as “watermark”; either transparent or opaque, if a watermark was placed over each page of the retracted article.

### Statistical analysis

We evaluated the distribution of all parameters qualitatively by plotting them as histograms. We reported discrete variables as counts or proportions and normally distributed continuous variables as means with standard deviations (SDs). We quantified inter-observer agreement for the reviewers’ assessments of article eligibility using Cohen’s kappa and interpreted values according to Landis and Koch as follows: 0, poor; 0.01 to 0.20, slight; 0.21 to 0.40, fair; 0.41 to 0.60, moderate; 0.61 to 0.80, substantial; and 0.81 to 1.00, almost perfect [18]. All statistical analyses were performed using Microsoft Excel (Santa Rosa, CA, USA, 2011).

## Results

### Identified retracted articles

Our search strategy yielded 1167 studies, 580 of which were excluded in the screening of titles and abstracts, followed by full-text screening. An additional 16 papers could not be accessed; thus, 571 retracted publications were included in the final analysis (Fig. 1). Inter-observer agreement between the reviewers for article inclusion was moderate (kappa 0.63, 95% CI 0.59 to 0.67)

**Fig. 1:**
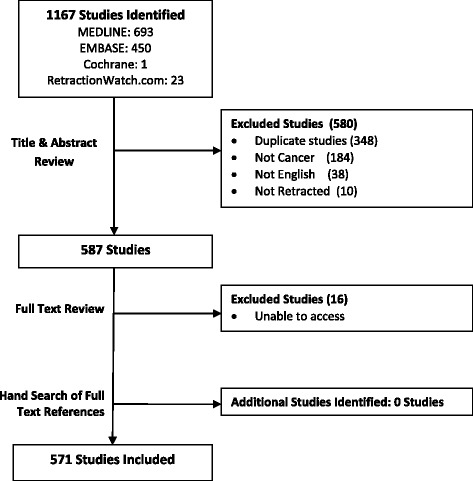
Identification of retracted articles

The majority of the retractions (374/571 [65.5%]) were basic science publications. Clinical studies accounted for 191/571 (33.4%) of the retractions and 6/571 (1.1%) were letters or commentaries (Fig. 2). A large range of study designs, from case reports to systematic reviews and meta-analyses, were identified in the retracted studies. The retracted clinical papers included 50 review papers, 7 meta-analyses, 23 randomized controlled trials, 64 observational studies, and 47 case series. Six authors were noted to have at least 5 retractions.

**Fig. 2:**
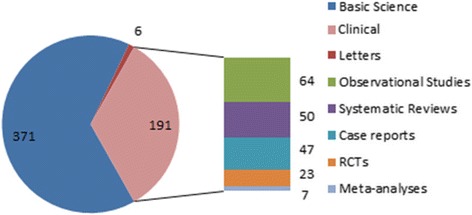
Classification of retracted papers

Retracted articles were identified in journals with impact factors ranging from 0 (discontinued) to 55.87. The average impact factor was 5.4 with a median of 3.54 and an interquartile range (IQR) of 1.8–5.5. On average, a retracted article was cited 44 times, with a range of 0–742, median of 18, and an IQR 6–51. Overall, 34 instances were found with a primary author having two distinct retracted publications. There were 8 instances of an author with three retracted publications, 4 instances of an author with four retractions, and 1 instance of an author with five retractions. There was one instance each of an author with seven and eight retracted publications, and two authors were found to each have nine retracted publications.

With respect to national affiliation, authors from the USA and China were 1st and 2nd in total number of retractions with 153 and 103 respectively. All countries with more than 10 retracted cancer publications are listed in Table 2.

**Table 2:** Number of retracted cancer publications by country

Country	Number of Retractions
USA	153
China	103
Japan	59
India	45
Germany	29
Italy	23
South Korea	17
UK	14

Country of the corresponding author; only countries with more than 10 retractions are included in the table

### Frequency of retracted articles

Given the time lag between publication and retraction, only two of the papers in our analysis were published in 2015. More than three quarters of all retracted publications in the cancer literature (436/571, 76.4%) occurred in the decade between 2005 and 2014. The preceding decade, 1995–2004, accounted for 95/571 (16.6%) of all retracted papers and the articles published prior to 1995 account for only 38/571 (6.7%) of all retracted papers in the cancer literature (Fig. 3). The average time between article publication and date of retraction was 2.3 years (SD 2.1) for papers published between 2005 and 2014, 7.3 years (SD 6.4) for papers published between 1995 and 2004, and almost 24 years (SD 6.7) for papers published prior to 1995. Our search for cancer retractions in a single database (MEDLINE) over each year from 2000 to 2010 indicates that the proportion of retracted cancer publications increased fivefold over that time period (Fig. 4, Table 3).

**Fig. 3:**
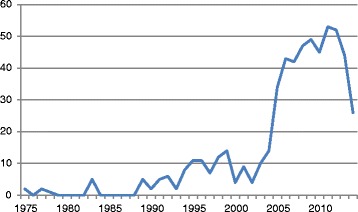
Annual number of retractions in cancer research publications. The number of retracted articles per year has rapidly increased. Due to the lag time between publication and retraction, only two retracted articles were identified in 2015 at the time of the literature search

**Fig. 4:**
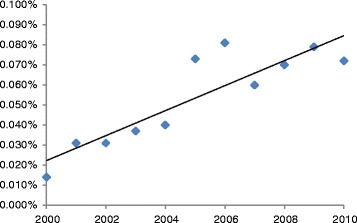
Proportion of retracted publications in relation to the total annual number of cancer research articles

**Table 3:** Reasons for retractions of publications in cancer research

Reason for retraction	Number	Percent
Duplicate publishing	162	28.4
Error	138	24.2
Fraud	104	18.2
Plagiarism	82	14.4
Authorship issues	22	3.9
Ethics issues	12	2.1
Not stated	56	9.8
Other	5	0.9
Total	571	100

### Reasons for retraction

The reasons for retraction are outlined in Table 3. Eighty-two retractions (14.4%) were attributed to plagiarism while 162 (28.4%) were attributed to the broader category of fraud. One hundred and four (18.2%) of the retractions were due to duplicate publication. Error on the part of the authors accounted for 138 retraction (24.2%). Authorship issues accounted for 22 retracted articles (3.9%). Ethical issues resulted in 12 retracted studies (2.1%). In 56 cases (9.8%), the reason for retraction was not stated. Often a statement such as “The authors wish to withdraw the paper and apologize for any inconvenience” was provided [19]. The remaining 5 papers were withdrawn because their methodology was based on retracted papers or because they were review articles largely based on retracted papers.

### Method of retraction notification

One hundred and sixty-seven (29.2%) of retracted studies were available online in their original intact form. The majority of retracted papers, 351/571 (61.5%), were available online but with a watermark over each page. The watermark was almost equally likely to be transparent (191 retractions) or opaque (160 retractions). Of the retracted articles, 53 (9.3%) had been permanently removed and were unavailable for download, with only the title of the article and the retraction notice hosted on the journal’s website.

## Discussion

### Summary of findings

As in other research fields, a vastly increased number of publications in cancer research were retracted in the past 10 years compared to any prior decade. Academic misconduct in the form of plagiarism, duplicate publication, and fraud accounts for 61% of retracted cancer publications. Error on the part of the authors resulted in 24.2% of the retracted studies. The reason for retraction was not stated in 9.8% of cases. Most of the retracted articles were still hosted online by the associated journal, but with a watermark stamped over each page. Retracted cancer publications are currently three times more likely to be available online in intact from, than to have been permanently removed. Reasons for retraction not directly related to academic misconduct such as authorship issues and novel research based on retracted work, together account for less than 5% of all retracted articles in the cancer literature.

### Limitations

While our search methodology aimed to be as inclusive as possible, we acknowledge that it may not have captured all retracted articles. MEDLINE has a specific filter for retracted articles, but other journal databases do not, and although our strategy tried to circumvent this by being as general as possible, there may have been some missed retracted articles.

The classification of the reason for retraction proved challenging, particularly when the retraction notice simply indicated ‘error’ on the part of the authors. It was often not further clarified and thus assumptions were made in these cases as to whether the error was accidental or intended. A significant proportion of retracted cancer papers, 24.2%, fall under the nebulous “error” category. While some retraction statements offered a specific error notice, such as the use of an erroneous cell line, this category may be used by journals as a euphemistic label for more nefarious reasons for retraction [20].

### Relation to previous literature

While the reasons for retractions were previously attributed to honest error [21], a 2012 study of retractions in all biomedical sciences showed that 67.4% of retractions were attributable to misconduct [22]. A 2016 study of retractions occurring from one journal publisher showed that academic misconduct may account for as much as 76% of all retractions. That rate includes plagiarism, fraud or attempted fraud, and duplicate publication. These findings are similar to those of our current study in which we determined that 61% of cancer retractions are due to research misconduct. Previous studies have also shown that retracted papers published after 2002 have a shorter time to retraction, averaging closer to 2 years, instead of a 4 year delay to retraction for papers published prior to 2002 [23]. The current study showed a similar trend towards significantly shorter intervals between cancer publication and cancer retraction over time.

To our knowledge, the current study is the first to characterize the state of retracted publications specifically within the field of cancer research. The academic pressures that are known to motivate scientific misconduct in other fields are certainly present in cancer research [24, 25]. A survey of 434 cancer research faculty and trainees at the MD Anderson Cancer Center revealed that greater than 50% had encountered the inability to reproduce data that was published elsewhere, at least once in their careers [26]. Perhaps more concerning is that this same study found that 31% of respondents “noted pressure from a mentor to prove his/her hypothesis correct, even though the data may not support the hypothesis” [26]. Moreover, 18.6% acknowledged feeling “pressured to publish findings of which you had doubt” [26].

Even established researchers with a career’s worth of foundational work can have subsequent work retracted. Dr Robert Weinberg and Dr Scott Valastyan have four shared cancer retractions from their time at Massachusetts Institute of Technology [27]. This is significant because Dr Weinberg discovered the first human oncogene (Ras) and tumor suppresor (Rb) in the 1990s [28, 29]. Dr Valastyan, the lead author on the retracted papers, was previously a recipient of a $156,000 Runyon cancer research award but has not published anything since 2012, when the retractions first came to light [27].

The clearest example of the deleterious effects that academic misconduct can have on cancer patients may come from Dr Anil Potti who was awarded a $729,000 research grant from the American Cancer Society while at Duke University, but was subsequently found to have falsified multiple research datasets [30]. The patients enrolled in prospective trials based on this fabricated data were given sub-optimal gene-targeted cancer therapy and this has led to multiple lawsuits [10]. Dr Potti no longer works at Duke, has not received NIH funding since 2010, and is required to have his research supervised until 2020 [10].

### Implications

The Committee on Publication Ethics (COPE) is an authoritative body for publishing ethics and has published several recommendations for journal editors regarding the retraction of publications. These include the publishing of a retraction notice with a link to the retracted article, clearly stating the reason for retraction and the responsible person(s), and marking the paper with a transparent watermark [31]. Our findings that 9.8% of retracted cancer articles are not accompanied by a retraction notice and that 29.2% of retracted papers are still available online in unaltered fashion, indicate that retracted articles in the cancer literature fall short of meeting COPE standards. The cancer research field is not alone in the struggle to maintain standards in retraction notices [32, 33]. Decullier et al. analyzed conformity to COPE guidelines for all retracted scientific articles published in 2008 and showed similar insufficient handling of retraction notices [34]. A study on retracted papers in the anesthesia literature described a similarly large proportion of intact retracted papers [35].

Our study raises awareness of this topic for journals, editors and peer reviewers in cancer research as well as highlights the importance of post-publication peer review in cancer research. Gasparyan et al. have shown that increased post-publication peer review due to digitization of research and open access journals may be responsible for the increased identification of errors and inconsistencies in research [36]. Furthermore, cancer journals should follow COPE and ICMJE guidelines for proper and clear reporting when dealing with possible retractions

## Conclusions

The number of retracted publications in the cancer literature is increasing rapidly, and cancer retractions are largely due to academic misconduct as opposed to honest error. Consequences to cancer patients and the cancer research community at large can be significant as invalid publication may have detrimental effect on patients treated in everyday practice. Despite the implications of this important issue, cancer journals fall short of the well-articulated COPE/ICMJE guidelines on the reporting of retractions [37, 38].

## Abbreviations

COPE: Committee on Publication Ethics
ICMJE: International Committee of Medical Journal Editors
IQR: Inter-quartile range
SD: Standard deviation

## References

[1] Atlas MC (2004). Retraction policies of high-impact biomedical journals. J Med Libr Assoc.

[2] Coats AJ (2009). Ethical authorship and publishing. Int J Cardiol.

[3] Redman BK, Yarandi HN, Merz JF (2008). Empirical developments in retraction. J Med Ethics.

[4] Cokol M, Ozbay F, Rodriguez‐Esteban R (2008). Retraction rates are on the rise. EMBO Rep.

[5] Van Noorden R (2011). The trouble with retractions. Nature.

[6] Grieneisen ML, Zhang M (2012). A comprehensive survey of retracted articles from the scholarly literature. PLoS One.

[7] Amos KA (2014). The ethics of scholarly publishing: exploring differences in plagiarism and duplicate publication across nations. J Med Libr Assoc.

[8] Stern AM (2014). Financial costs and personal consequences of research misconduct resulting in retracted publications. Elife.

[9] Fanelli D (2013). Why growing retractions are (mostly) a good sign. PLoS Med.

[10] Pelley S (2016). Deception at Duke: fraud in cancer care? 2012 June 15.

[11] Steen RG (2011). Retractions in the medical literature: how many patients are put at risk by flawed research?. J Med Ethics.

[12] Oranksy, I. Top 10 most highly cited retracted papers. 2015 June 15, 2016]; Available from: http://retractionwatch.com/the-retraction-watch-leaderboard/top-10-most-highly-cited-retracted-papers/.

[13] Mills EJ (2007). Epidemiology and reporting of randomized trials employing re-randomization of patient groups: a systematic survey. Contemp Clin Trials.

[14] Higgins JP, Green, S. Cochrane handbook for systematic reviews of interventions. Vol. 5. 2008: Wiley Online Library.

[15] Moher D (2009). Preferred reporting items for systematic reviews and meta-analyses: the PRISMA statement. Ann Intern Med.

[16] Burnham JF (2006). Scopus database: a review. Biomedical digital libraries.

[17] Fang FC, Casadevall A (2011). Retracted science and the retraction index. Infect Immun.

[18] Sim J, Wright CC (2005). The kappa statistic in reliability studies: use, interpretation, and sample size requirements. Phys Ther.

[19] Wang S, Wang Z (2013). Epigenetic aberrant methylation of tumor suppressor genes in small cell lung cancer. Journal of thoracic disease.

[20] Casadevall A, Steen RG, Fang FC (2014). Sources of error in the retracted scientific literature. FASEB J.

[21] Steen RG (2011). Retractions in the scientific literature: do authors deliberately commit research fraud?. J Med Ethics.

[22] Fang FC, Steen RG, Casadevall A (2012). Misconduct accounts for the majority of retracted scientific publications. Proc Natl Acad Sci.

[23] Steen RG, Casadevall A, Fang FC (2013). Why has the number of scientific retractions increased?. PLoS One.

[24] Moses H (2015). The anatomy of medical research: US and international comparisons. JAMA.

[25] Kornfeld DS (2012). Perspective: research misconduct: the search for a remedy. Acad Med.

[26] Mobley A (2013). A survey on data reproducibility in cancer research provides insights into our limited ability to translate findings from the laboratory to the clinic. PLoS One.

[27] Palus, S. Cancer Research retraction is fifth for Robert Weinberg; fourth for his former student. 2015 June 15, 2016]; Available from: http://retractionwatch.com/2015/07/06/cancer-research-retraction-is-fifth-for-robert-weinberg-fourth-for-his-former-student/.

[28] Egan SE (1993). Association of Sos Ras exchange protein with Grb2 is implicated in tyrosine kinase signal transduction and transformation. Nature.

[29] Weinberg RA (1995). The retinoblastoma protein and cell cycle control. Cell.

[30] It’s official: Anil Potti faked cancer research data, say Feds. 2015 June 15th, 2016]; Available from: http://retractionwatch.com/2015/11/07/its-official-anil-potti-faked-data-say-feds/.

[31] (COPE), C.o.P.E., Code of conduct and best practice guidelines for journal editors. publicationethics.org, 2011: p. 1-12.

[32] Marcus A, Oransky I (2014). What studies of retractions tell us. J Microbiol Biol Educ.

[33] Wager E, Williams P (2011). Why and how do journals retract articles? An analysis of Medline retractions 1988–2008. J Med Ethics.

[34] Decullier E (2013). Visibility of retractions: a cross-sectional one-year study. BMC Res Notes.

[35] Elia N, Wager E, Tramer MR (2014). Fate of articles that warranted retraction due to ethical concerns: a descriptive cross-sectional study. PLoS One.

[36] Gasparyan AY (2014). Self-correction in biomedical publications and the scientific impact. Croat Med J.

[37] Editors, I.C.o.M.J., International Committee of Medical Journal Editors (ICMJE): uniform requirements for manuscripts submitted to Biomedical Journals: writing and editing for biomedical publication*.* Haematologica. 2004;89(3):264 15020262

[38] Graf C (2007). Best practice guidelines on publication ethics: a publisher's perspective. Int J Clin Pract.

